# Identification of stable pollen development related reference genes for accurate qRT-PCR analysis and morphological variations in autotetraploid and diploid rice

**DOI:** 10.1371/journal.pone.0253244

**Published:** 2021-06-29

**Authors:** Jinwen Wu, Hao Fan, Yifan Hu, Haibin Guo, Hong Lin, Yinzhi Jiao, Zijun Lu, Susu Du, Xiangdong Liu, Muhammad Qasim Shahid

**Affiliations:** 1 State Key Laboratory for Conservation and Utilization of Subtropical Agro-Bioresources, South China Agricultural University, Guangzhou, China; 2 Guangdong Provincial Key Laboratory of Plant Molecular Breeding, South China Agricultural University, Guangzhou, China; 3 College of Agriculture, South China Agricultural University, Guangzhou, China; Nigde Omer Halisdemir University, TURKEY

## Abstract

Autotetraploid rice exhibited hybrid vigor and greater genetic variation compared to diploid rice, but low pollen fertility is a major hindrance for its utilization. Our previous analysis revealed that large number of pollen fertility genes were exhibited down-regulation in autotetraploid rice. Hence, it is of utmost importance to reveal the expression patterns of pollen fertility genes with high accuracy. To find stable reference genes for autotetraploid rice, we compared the pollen development stages between diploid and autotetraploid rice, and 14 candidate genes were selected based on transcriptome analysis to evaluate their expression levels. Autotetraploid rice (i.e. Taichung65-4x) displayed lower seed set (40.40%) and higher percentage of abnormalities during the pollen development process than its diploid counterpart. To detect the candidate reference genes for pollen development of autotetraploid and diploid rice, we used five different algorithms, including NormFinder, BestKeeper, ΔCt method, geNorm and Re-Finder to evaluate their expression patterns stability. Consequently, we identified two genes, *Cytochrome b5* and *CPI*, as the best candidate reference genes for qRT-PCR normalization in autotetraploid and diploid rice during pre-meiosis, meiosis, single microspore and bicellular pollen development stages. However, *Cytochrome b5* was found to be the most stably expressed gene during different pollen development stages in autotetraploid rice. The results of our study provide a platform for subsequent gene expression analyses in autotetraploid rice, which could also be used in other polyploid plants.

## Introduction

Autotetraploid rice is a new germplasm derived from diploid rice by chromosomes doubling. Compared with its corresponding diploid rice, several advantages, including the higher 1000-grain weight, larger grain size, longer panicles and stronger stem, have been noticed that in autotetraploid rice [1–3]. In spite of the noticeable alterations in polyploidy rice at cytological and morphological levels, low pollen fertility is still the primary hindrance in the application of tetraploid rice [4–6]. Consequently, it is of great demand to reveal the cause of low pollen sterility in tetraploid rice. In the case of autotetraploid rice, microarrays and RNA-seq analysis have been used to reveal the large-scale expression analysis [7–9], and quantitative reverse transcription PCR (qRT-PCR) analysis is one of the major choices for measuring expression patterns of selected genes with high precision. Large numbers of differentially expressed genes displayed down-regulation in autotetraploid rice compared with diploid rice [7–9]. Although the procedure of qRT-PCR analysis offers high reproducibility, precision and throughput [10], the accuracy of this method depends on several factors, such as selection of appropriate reference genes [11–13]. Therefore, it is necessary to detect the reference gene with stable expression level as an internal reference gene to reduce the error between samples in autotetraploid rice.

The reference genes used in qRT-PCR analysis are mainly derived from two sources: new candidate genes detected by transcriptome analysis for stable expression patterns across various experimental conditions, and genes selected from conventional designated housekeeping genes or their homologs. Reference genes, including *ACT11*, *GAPDH*, *UBQ5* and *18s rRNA*, are commonly applied as candidate reference genes for normalization of qRT-PCR data [14]. The qRT-PCR validation of reference genes have been described in many plant species, for example, *EF1a* and *UBQ5* displayed the most stable expression patterns in 25 rice samples that were isolated from various tissues at different development stages in rice. However, previous studies demonstrated that several of these commonly reference genes didn’t show stable expression in different tissues, experimental conditions and development stages [14–16]. Non-stable reference genes could result in errors in experiments under different conditions. For example, *ACT1* expression levels are unstable during various developmental stages [15], though it is a suitable reference gene in rice during seed development [17]. *GAPDH* display stable expressions in cultivated rice germinating seeds under hypoxia [18], but unstable results were generated by *GAPDH* in an iron toxicity study of rice [19]. Consequently, a stable rapid approach is required to find excellent reference genes for the systematic validation of expression levels in different plant tissues under different experimental conditions.

In recent years, to understand the genetic regulation of development process, transcriptome analysis is being used frequently. Moreover, numerous gene expression datasets have been publicly available on TIGR (Rice Genome Annotation Project Database), GEO (Gene Expression Omnibus Database), and Rice XPro (Rice Expression Profile Database) [20, 21]. The stable development genes and reliable development network could be identified and designated as the candidate reference genes. For example, a recent study detected novel reference genes by transcriptome analysis from different rice tissues and development stages [22]. Several research works have been conducted on the pollen development process in autotetraploid rice. Significant expression differences were detected in pollen development stage between the autotetraploid and its diploid rice, and 42 meiosis meiosis-related or stage-specific genes showed differential expression patterns by transcriptome analysis [7]. Moreover, 75 meiosis-related genes were found to be differentially expressed in T449-4x compared to its original diploid rice during meiosis stage [8].

Reference gene is the critical factor for gene expression analysis and it must show moderate and stable expression levels in different cell tissues and experimental conditions [18, 22, 23]. Several methods have been used to screen and evaluate candidate reference genes from different experimental datasets. Several programs were used to measure expression stabilities of these genes, such as geNorm [11], delta Ct (ΔCt) method [11], RefFinder [24], BestKeeper [12] and NormFinder [25]. Among these programs, operating principle of the geNorm program is similar to that of NormFinder, but the former can select the optimal number of reference genes. In contrast to both these programs, BestKeeper program can detect the stability of both target and reference genes. RefFinder is a computational tool and considered as a complete evaluation package and offers information using data from BestKeeper, geNorm, and NormFinder, which detect a stable single gene or gene combination as the internal control [24].

In the present study, we investigated the phenotypic variations in morphological traits and compared the pollen development process between autotetraploid and diploid rice. Moreover, we selected 14 candidate reference genes, including 7 commonly used housekeeping genes and 7 stably expressed genes based on the transcriptome data, to assess their expression stability for qRT-PCR analysis during pollen development in polyploid and diploid rice. Moreover, the expression patterns of six target genes were examined to demonstrate the effectiveness of the selected reference genes in this study. The results of the present study will be valuable for future research to assess the expression patterns of target genes by using these reference genes during pollen development in autotetraploid rice, and also provide the evidence for the selection of reference genes in polyploid plants.

## Materials and methods

### Plant material and analysis of morphological traits

Two lines, Taichung65-4x and its corresponding diploid rice, Taichung65-2x, were used in this study. Taichung65-4x is an autotetraploid rice material derived from the diploid rice by chromosomes doubling. To evaluate the phenotypic variations between Taichung65-4x and its corresponding diploid rice, a total of nine morphological traits, i.e., panicle length (PL, cm), plant height (PH, cm), flag leaf length (FL, cm), number of effective panicles, flag leaf width (FW, cm), grain width (GW, cm), grain length (GL, cm), grain length to width ratio and seed set (100%), were selected.

### Pollen fertility, WE-CLSM analysis and semi-thin section analysis

The pollen fertility of Taichung65-4x and its corresponding diploid rice was investigated according to our previous study [26]. Spikelets of Taichung65-4x and its corresponding diploid rice were collected 1 or 2 d preanthesis and fixed in Carnoy’s solution for 24 hours. Then the samples were kept in 70% alcohol. Three spikelets were randomly selected from each plant, and six anthers from one spikelet were stained with 1% (wt/vol) iodine potassium iodide (I_2_–KI) solution on a glass slide. More than 1000 pollen grains were counted to estimate pollen fertility under a microscope (Motic BA200).

Microgametogenesis observation was conducted according to our previous study [7]. The inflorescences were collected during pollen development process and kept in petri dish with a moist paper. Anthers were removed from the floret and squashed with the forceps onto the glass slide, and then a small drop of 10 mg/L eosin B (C_20_H_6_N_2_O_9_Br_2_Na_2_, FW 624.1) solution (dissolved in 4% sucrose) was added, and observed under Leica SPE laser scanning confocal microscope (Leica Microsystems, Heidelberg, Germany). Excitation wave length was 543 nm, and emission light was noticed between 550 and 630 nm [27].

Semi-thin section analysis was performed to detect the pollen development variations between Taichung65-4x and its corresponding rice [28]. The anthers were collected during different development stages and fixed in FAA solution for 48 hours. After washing in 50% ethanol several times, samples were dehydrated in a series of ethanol solutions and then embedded by a Leica 7022 historesin embedding kit (Leica, Nussloch, Germany) according to its manufacturer. Embedded samples were further sectioned using the Leica RM2235 manual rotary microtome and stained with 1% toluidine blue and sealed with neutral balsam.

### Total RNA isolation and cDNA synthesis

Anthers of different pollen development stages were collected from pre-meiotic stage to bicellular stage and stained with 1% (wt/vol) acetocarmine and confirmed by the microscope according to our previous study [7]. The classification of anther development stages in diploid and autotetraploid rice according to our previous study with some minor modifications (S1 Table in S1 File). After collection, total RNA from anthers in differential pollen development stage were extracted by TRIzol Reagent. Three biological replicates per samples were stored and kept at -80°C until RNA extraction. RNA quality was quantitated by spectrophotometer and assessed by formaldehyde agarose gel electrophoresis [7]. RNA samples with a 260/280 ratio between 1.8 and 2.1 were selected for reverse transcription. Equal quantities (1μg) of total RNA were immediately transcribed into cDNA using PrimeScript^™^ RT reagent Kit with gDNA Eraser (Takara) according to the manufacturer protocol.

### Selection of candidate reference genes and primer designing

A total of 14 potential reference genes were selected, including the seven reported potential candidates and seven candidate reference genes using the dataset and combined with our autotetraploid rice transcriptome dataset (Table 1). Primers were designed based on the sequences of the 14 genes using Primer3 plus (http://bioinfo.ut.ee/primer3-0.4.0/primer3/) with the following criteria: GC content 45–65%, optimal Tm 58–61°C, primer length 18-22bp, and amplicon length 80-150bp (Table 1). The feasibility of 14 pairs was preliminarily verified by standard qRT-PCR using Premix Ex Taq (TaKara, Dalian, China), and each gene was verified by 2% agarose gel electrophoresis and sequenced to ensure its reliability.

**Table 1:** Description of the candidate reference genes, primer sequence and qRT-PCR amplification efficiencies.

Gene symbol	Gene ID	Primer sequences (5’-3’)		Amplification Length	Amplification efficiency
		Forward	Reverse		
*OsActin1*	LOC_Os03g50885	TGGCATCTCTCAGCACATTCC	TGCACAATGGATGGGTCAGA	76	99.4
*UBQ5*	LOC_Os01g22490	ACCACTTCGACCGCCACTACT	ACGCCTAAGCCTGCTGGTT	69	96.3
*OsAOC*	LOC_Os03g32314	GAGGCTTCTTGGTAGTAGGTGGA	CGTAGTGGCGGTCGTTGTAGT	79	91.2
*β-TUB*	LOC_Os01g59150	GCTGACCACACCTAGCTTTGG	AGGGAACCTTAGGCAGCATGT	82	95.6
*GAPDH*	LOC_Os08g03290	GTCTGCATCAGAGGGAAAG	AGAGCAATTCCAGCCTTGG	120	91.4
*CPI*	LOC_Os01g05490	TAACTGGTGCGAACTGCAAG	CGGAGTTGATGATGTCGATG	105	98.1
*EF1a*	LOC_Os03g08020	TTTCACTCTTGGTGTGAAGCAGAT	GACTTCCTTCACGATTTCATCGTAA	103	92.6
*SRP*	LOC_Os08g37444	GCTCATCAATCTCAATAG	TATCAATCTGGTAGTAGG	136	102.1
*RIC1*	LOC_Os01g37800	GATAGGTATGCTAGTGAA	GTATTCCAATCTCATCAG	118	101.7
*PFP*	LOC_Os03g50480	CCATTATTATACACGATATGACTA	AACATCTGACCGAATCTC	133	99.6
*FLO16*	LOC_Os10g33800	AAGGAAGGATGTTATGTCAA	ATTGGCAACTACCAGAAC	103	103.3
*Cytochrome b5*	LOC_Os05g01820	TGATGGATGAGTATTACG	AGGATCTTGATGATGAAC	124	102.1
*OsATG8a*	LOC_Os07g32800	TTGAGAAGGCTGACAAGA	TTCCGAACCACATAGACA	100	93.4
*CPuORF7*	LOC_Os04g42090	ATTGACTCTGTTCTGGAT	AGGTCTTAATCACAATCTTAT	127	104.2

### Quantitative reverse transcription PCR analysis

The selected reference genes of Taichung 65-4x and its corresponding diploid rice during pollen development were screened by qRT-PCR analysis. The amplification reaction was performed on the Lightcycler480 system (Roche) using the Advanced SYBR Green Supermix Kit (Bio-RAD). Total volume of reaction system was 20μL, including the mixture 10μL, 0.5μL each primer (10μM), cDNA 1μL and ddH_2_O 8.0μL. The qRT-PCR cycles were performed using the following reaction conditions: 95°C for 30s, 40 cycles of 95°C denaturation for 5s and 58°C annealing and extension for 20s. All qRT-PCR reactions were performed in triplicate and three biological repeats were performed at each pollen development stage.

### Analysis of expression levels of candidate reference genes

Five differential algorithms: delta Ct (ΔCt) method [11], geNorm [11], NormFinder [25], RefFinder (https://www.heartcure.com.au/reffinder/?type=reference) and BestKeeper [12] were used to assess the stability of 14 potential reference genes. The geNorm program calculates the M value based on the variation of each candidate reference gene. The highest M value represents the least stable gene under a given experimental treatment, while the lowest M value shows the most stable reference gene. Delta-Ct (ΔCt) technique is similar to geNorm program that can be executed using an excel sheet, and it calculates the differential ΔCt value of candidate reference genes in which small value indicates the stability of gene transcript. NormFinder takes both intragroup and intergroup differences into account and calculates the stability value. The candidate reference gene having the lowest value indicates the highest stability. BestKeeper method is based on the standard deviation (SD) and coefficient of variance (CV). The genes with least SD value representing the highest stability. RefFinder combined the data from the geNorm (M value), Delta-Ct (ΔCt value), NormFinder (stability value) and BestKeeper (CV and SD), and identify the most stable genes by computing the geometric mean for each gene. The genes with the lowest values are designated as the most stable.

### Validation of reference genes for qRT-PCR normalization

To validate the expression patterns of stable genes indicated by the five algorithms, two least stable and two most stable genes were used for normalization of qRT-PCR data of six pollen development related genes. To confirm the reliability of reference genes, six pollen development related genes were selected from the previous studies [7, 9]. The qRT-PCR results were calculated using the 2^-ΔΔCt^ method [29], and all reactions were executed in triplicate.

## Results

### Comparison of phenotypic traits between Taichung65-4x and its diploid counterpart

**Fig 1:**
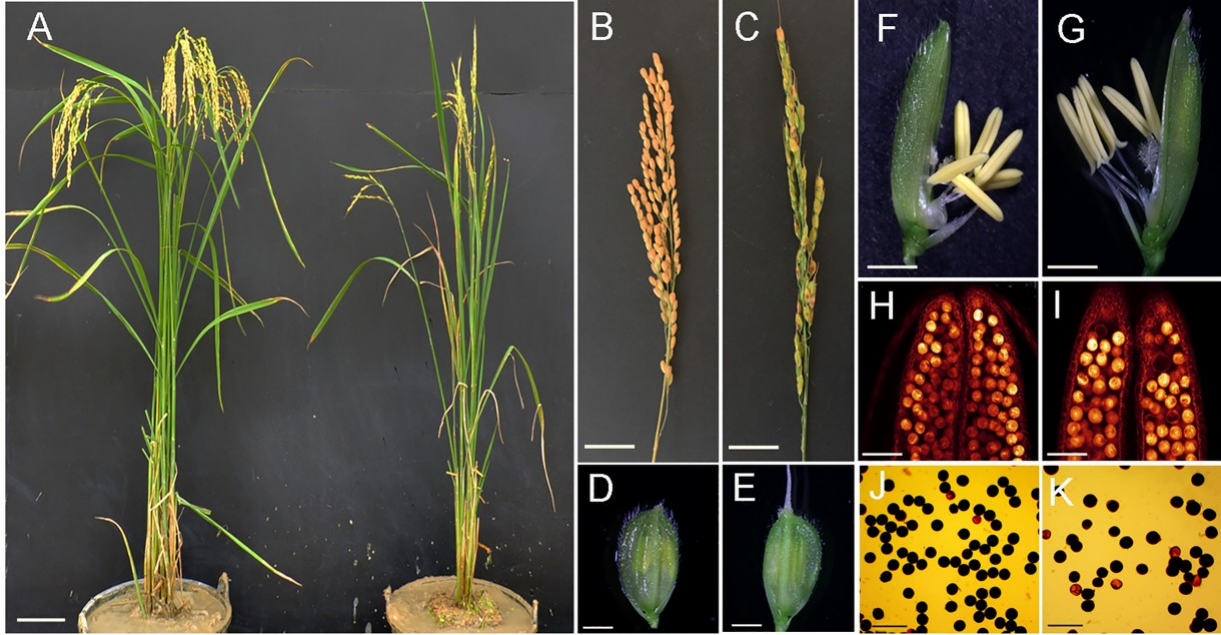
Phenotypic characterization of Taichung65-4x and its corresponding diploid rice. (A) Morphologies of Taichung65-4x and Taichung65-2x. Bars = 10 cm. (B) and (C) Panicles of Taichung65-4x and Taichung65-2x. Bars = 2 cm. (D) and (E) Grain size of Taichung65-4x and Taichung65-2x. Bars = 1 mm. (F) and (G) Floral organs of Taichung65-4x and Taichung65-2x. Bars = 1 mm. (H) and (I) Anthers of Taichung65-4x and Taichung65-2x were observed by using the WE-CLSM (Whole-mount eosin B-staining confocal laser scanning microscopy). Bars = 100 μm. (J) and (K) Pollens of Taichung65-4x and Taichung65-2x, respectively. Bars = 100 μm.

To evaluate the phenotypic variations between Taichung65-4x and its corresponding diploid rice, we compared the morphological traits between Taichung65-4x and its diploid counterpart. From this study, phenotypic changes in Taichung 65-4x, such as plant height, number of effective panicles, flag leaf length, and seed set, were significantly different from the diploid rice (S2 Table in S1 File). The average values of plant height, number of effective panicles, flag leaf length and seed set in autotetraploid rice were 9.83%, 44.45%, 13.23% and 50.79% lower than diploid rice, respectively. However, numbers of effective panicles per plant, pollen fertility and plant height in autotetraploid rice were lower than the diploid rice. Altogether, Taichung 65-4x had shown markedly different morphological traits compared with the diploid rice (Fig 1).

### Comparison of pollen development process between Taichung65-4x and its corresponding rice

To evaluate the pollen development process between Taichung65-4x and its corresponding rice, we observed the anther and pollen development stages using the semi-thin section and WE-CLSM (Whole-mount eosin B-staining confocal laser scanning microscopy) analysis. Anther development in Taichung65-4x was similar to that of its corresponding diploid rice, and different types of abnormalities were observed in Taichung65-4x during its anther development (S1 Fig in S1 File).

**Fig 2:**
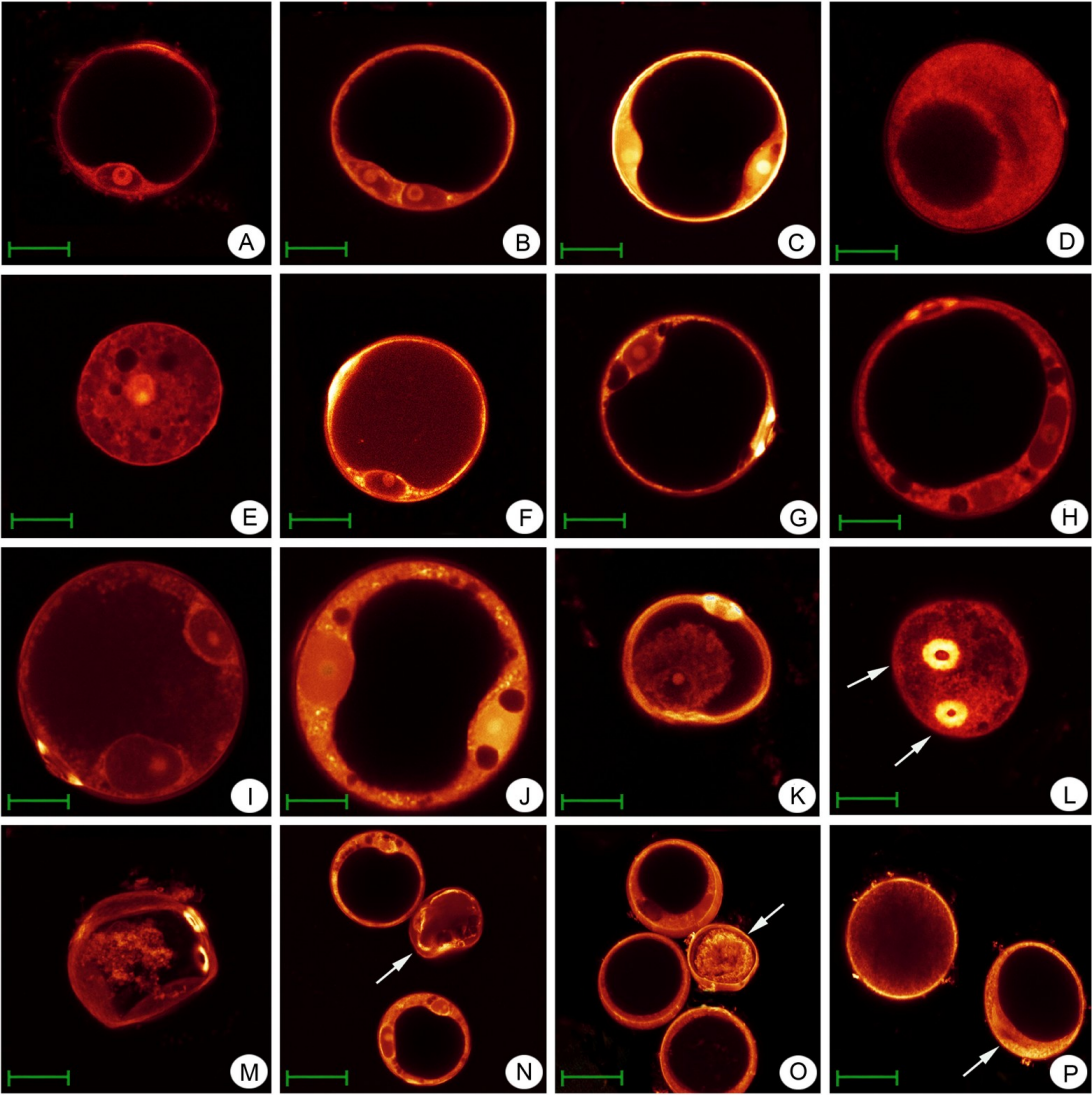
Microgametogenesis of Taichung65-4x and its corresponding diploid rice. (A-D) Microgametogenesis of Taichung65-2x. (A) Late microspore stage. (B) Early bicellular pollen stage. (C) Late bicellular pollen stage. (D) Mature pollen stage. (E-P) Microgametogenesis of Taichung65-4x. (E) Middle microspore stage, vacuolization. (F) Late microspore stage. (G) Late microspore stage. (H) Early bicellular pollen stage. (I) Middle bicellular pollen stage. (J) Late bicellular pollen stage. (K) Middle microspore stage, cell shrinkage. (L) Late bicellular pollen stage, multiple apertures. (M) Late bicellular pollen stage, cell shrinkage and multiple apertures. (N) Late bicellular pollen stage, arrow indicate the cell shrinkage. (O) Middle bicellular pollen stage, arrow indicate the cell shrinkage. (P) Mature pollen stage, arrow indicate the asynchronous pollens. Bars = 40μm.

We further observed the microgametogenesis of Taichung65-4x and its corresponding diploid rice (Fig 2). Microgametogenesis of diploid rice (Fig 2A–2D) and autotetraploid rice, Taichung65-4x was almost similar (Fig 2E–2J). Microgametogenesis in Taichung 65-4x could be divided into seven stages, and its process was similar to original diploid rice. However, various abnormal microspores in microgametogenesis was also detected in Taichung65-4x (Fig 2K–2P). In microgametogenesis of Taichung 65-4x, following major abnormal microspores were commonly detected, such as microspores degeneration (Fig 2K), multi-aperture (Fig 2L), cytoplasmic shrinkage (Fig 2M), and cell abnormalities in bicellular pollen stage (Fig 2N and 2O) and mature pollen stage (Fig 2P). All of these results indicated that the pollen development of autotetraploid rice was significantly different compared to its corresponding diploid rice.

### Identification of candidate reference genes for the pollen development based on autotetraploid rice transcriptome data

**Fig 3:**
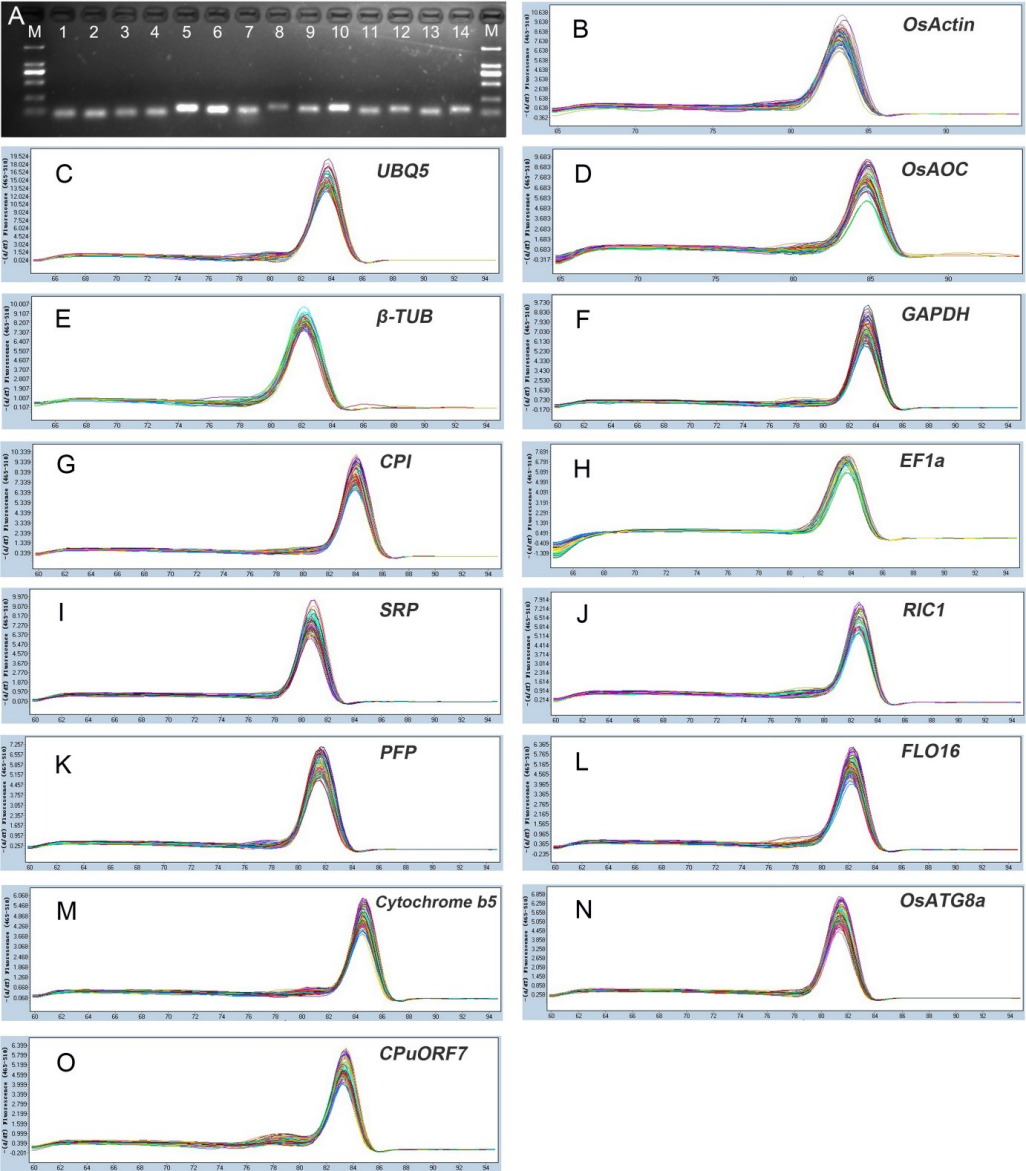
Specificity of primers and its amplification size. (A) Amplification of PCR products for 14 genes in agarose gel (1.5%) electrophoresis. (M: DNA Marker 2000bp; lanes 1–14: *OsActin1*, *UBQ5*, *OsAOC*, *β-TUB*, *GAPDH*, *CPI*, *EF-1a*, *SRP*, *RIC1*, *PFP*, *FLO16*, *Cytochrome b5*, *OsATG8a* and *CPuORF7*, respectively. (B to O) Melting curves of 14 candidate reference genes show single peaks.

To select the candidate reference genes for pollen development process in autotetraploid rice, we screened the expression patterns of autotetraploid rice and selected 14 candidate reference genes (Table 1). Among these candidate reference genes, seven candidate reference genes, including *OsActin1*, *UBQ5*, *OsAOC*, *β-TUB*, *GAPDH*, *CPI*, *EF-1a*, were commonly used reference genes and seven candidate reference genes (*SRP*, *RIC1*, *PFP*, *FLO16*, *Cytochrome b5*, *OsATG8a* and *CPuORF7*) were selected from the expression patterns of autotetraploid rice (Fig 3). We created a heat map based on the expression patterns of these 14 genes in autotetraploid rice during the different pollen development stages. The transcript levels of candidate reference genes displayed high stability than the common reference genes at different pollen development stages (S2 Fig in S1 File).

The cycle threshold (Ct) is further used to reflect the transcriptional stability of candidate reference genes (S3 Fig in S1 File). Here, we selected 14 candidate reference genes to detect its expression levels in diploid and autotetraploid rice during pollen development process. The expression pattern of each candidate reference gene is summarized in S3 Fig and S3 Table in S1 File. Among these candidate reference genes, Ct values in different pollen developmental stages ranged from 9.89 to 34.24. *OsActin1* exhibited the highest expression level, with a mean Ct of 13.75, and *OsAOC* showed the lowest expression pattern, with a mean Ct of 31.86. The coefficients of variation (CV) reflect the degree of deviation in the observed values within a sample (high values indicate higher variability). Among all comparison groups, *OsActin1* was found as the most variable (CV, 16.11%), while *Cytochrome b5* displayed the lowest variability with a CV value of 5.48% (S3 Table in S1 File).

### Transcription stability analyses of candidate reference genes

#### ΔCt method

ΔCt method was used to evaluate the stability of 14 candidate reference genes. Lower values of average standard deviations indicate the higher stability of candidate reference genes, which is suitable for qRT-PCR analysis. The results of the ΔCt method are reported in Table 2. In this study, *Cytochrome b5* was the most stable gene for all the samples, including total samples group, diploid rice group, autotetraploid rice group, MA stage and BCP stage, and the values of average standard deviations in each group were 1.44, 1.48, 1.30, 1.41 and 0.95, respectively. *CPuORF7* and *CPI* was the most stable gene for PMA, MA and SCP stage, respectively. Additionally, *Cytochrome b5* displayed the highest stability among the candidate reference genes.

**Table 2:** Stability analysis of candidate reference genes assayed by ΔCt method.

Rank	Total	Diploid	Autotetraploid	PMA	MA	SCP	BCP
1	*Cytochrome b5* (1.44)	*Cytochrome b5* (1.48)	*Cytochrome b5* (1.30)	*CPuORF7* (1.38)	*Cytochrome b5* (1.41)	*CPI* (0.84)	*Cytochrome b5* (0.95)
2	*CPI* (1.55)	*SRP* (1.49)	*OsATG8a* (1.41)	*CPI* (1.38)	*SRP* (1.43)	*GAPDH* (0.84)	*PFP* (0.96)
3	*SRP* (1.57)	*GAPDH* (1.51)	*CPI* (1.42)	*Cytochromeb5* (1.40)	*PFP* (1.44)	*Cytochrome b5* (0.86)	*SRP* (0.98)
4	*OsATG8a* (1.58)	*CPuORF7* (1.53)	*PFP* (1.45)	*PFP* (1.41)	*OsAOC* (1.48)	*PFP* (0.87)	*OsATG8a* (1.01)
5	*GAPDH* (1.59)	*CPI* (1.54)	*CPuORF7* (1.50)	*SRP* (1.46)	*EF1a* (1.48)	*OsATG8a* (0.88)	*RIC1* (1.05)
6	*PFP* (1.60)	*FLO16* (1.57)	*GAPDH* (1.55)	*GAPDH* (1.53)	*β-TUB* (1.48)	*RIC1* (0.89)	*EF1a* (1.05)
7	*CPuORF7* (1.64)	*OsATG8a* (1.60)	*SRP* (1.56)	*FLO16* (1.53)	*FLO16* (1.48)	*EF1a* (0.89)	*FLO16* (1.09)
8	*FLO16* (1.66)	*PFP* (1.61)	*RIC1* (1.62)	*RIC1* (1.74)	*CPI* (1.62)	*FLO16* (1.02)	*CPuORF7* (1.15)
9	*RIC1* (1.77)	*RIC1* (1.82)	*EF1a* (1.63)	*EF1a* (1.74)	*GAPDH* (1.64)	*SRP* (1.06)	*CPI* (1.17)
10	*EF1a* (1.78)	*EF1a* (1.84)	*FLO16* (1.64)	*OsATG8a* (1.83)	*OsActin1* (1.72)	*OsActin1* (1.20)	*GAPDH* (1.29)
11	*OsAOC* (2.44)	*β-TUB* (2.25)	*OsAOC* (1.87)	*UBQ5* (2.19)	*CPuORF7* (1.83)	*CPuORF7* (1.22)	*OsActin1* (1.42)
12	*β-TUB* (2.48)	*UBQ5* (2.80)	*OsActin1* (2.28)	*β-TUB* (2.33)	*UBQ5* (1.85)	*UBQ5* (1.37)	*UBQ5* (1.56)
13	*OsActin1* (2.68)	*OsAOC* (2.86)	*β-TUB* (2.35)	*OsActin1* (2.86)	*RIC1* (2.28)	*β-TUB* (1.58)	*OsAOC* (1.67)
14	*UBQ5* (2.75)	*OsActin1* (2.98)	*UBQ5* (2.50)	*OsAOC* (3.46)	*OsATG8a* (2.52)	*OsAOC* (1.63)	*β-TUB* (3.22)

Note: Total indicate the all samples of diploid and autotetraploid rice. Diploid indicate the all samples of diploid rice; Auto indicate the all samples of autotetraploid rice; PMA indicate the samples of pre-meiosis stage in diploid and autotetraploid rice; MA indicate the samples of meiosis stage in diploid and autotetraploid rice; SCP indicate the samples of single microspore stage in diploid and autotetraploid rice; BCP indicate the samples of bicellular pollen stage in diploid and autotetraploid rice.

#### GeNorm analysis

**Fig 4:**
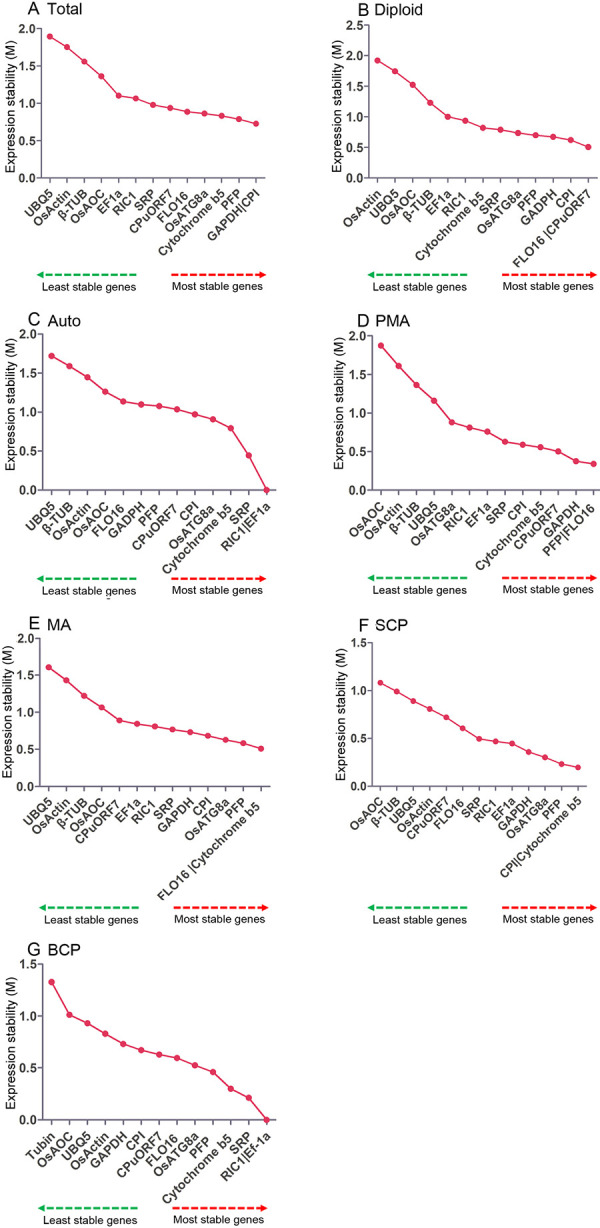
Stability analysis of candidate reference genes using the geNorm software. (A) All samples of autotetraploid and diploid rice. (B) All samples of diploid rice. (C) All samples of autotetraploid rice. (D) Samples of pre-meiosis stage in diploid and autotetraploid rice. (E) Samples of meiosis stage in diploid and autotetraploid rice. (F) Samples of single microspore stage in diploid and autotetraploid rice. (G) Samples of bicellular pollen stage in diploid and autotetraploid rice. Total indicate the all samples of diploid and autotetraploid rice. Diploid indicate the all samples of diploid rice; Auto indicate the all samples of autotetraploid rice; PMA indicate the samples of pre-meiosis stage in diploid and autotetraploid rice; MA indicate the samples of meiosis stage in diploid and autotetraploid rice; SCP indicate the samples of single microspore stage in diploid and autotetraploid rice; BCP indicate the samples of bicellular pollen stage in diploid and autotetraploid rice.

GeNorm program was used to evaluate the stability of 14 candidate reference genes using the M-values (Fig 4). The results of M value analyses demonstrated that *GAPDH* and *CPI*, which had the same values, were the most stable reference genes for all the samples (Fig 4A). *FLO16* and *CPuORF7* were the most stable reference genes for diploid rice (Fig 4B), while *RIC1* and *EF1a* were the most stable reference genes for all autotetraploid rice samples during different pollen development stages (Fig 4C). Among the different pollen development stages, *FLO16* and *PFP* were the most stable reference genes for PMA stage (Fig 4D), *FLO16* and *Cytochrome b5* were the most stable reference genes for MA stage (Fig 4E), *Cytochrome b5* and *CPI* were the most stable reference genes for SCP stage (Fig 4F), *RIC1* and *EF1a* were the most stable reference genes for BCP stage (Fig 4G). *UBQ5*, *OsActin1*, *OsAOC* and *β-TUB* were found to be the least stable reference genes for most of the samples.

**Fig 5:**
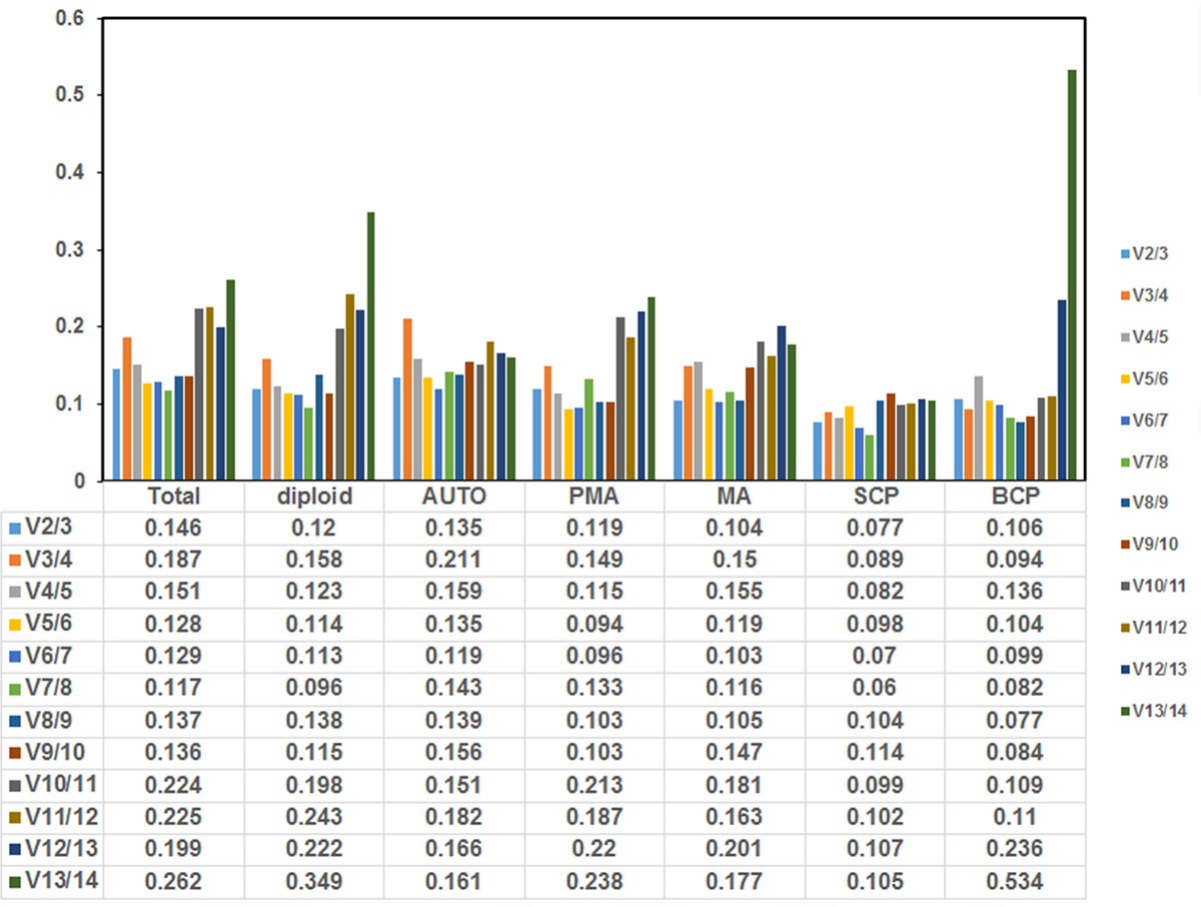
Determination of the optimal number of reference genes for normalization by pairwise variation (V) using geNorm software. The average pairwise variations (Vn/Vn+1) were analyzed to measure the effect of adding reference gene in the qRT-PCR. Total indicate the all samples of diploid and autotetraploid rice. Diploid indicate the all samples of diploid rice; Auto indicate the all samples of autotetraploid rice; PMA indicate the samples of pre-meiosis stage in diploid and autotetraploid rice; MA indicate the samples of meiosis stage in diploid and autotetraploid rice; SCP indicate the samples of single microspore stage in diploid and autotetraploid rice; BCP indicate the samples of bicellular pollen stage in diploid and autotetraploid rice.

Moreover, pairwise difference (Vn/Vn+1) between two normalization factors was estimated by the geNorm software to calculate the optimum number of reference genes for precise normalization (Fig 5). A cut-off value of 0.15 (Vn value) is a default threshold demonstrating that an extra reference gene has no effect on normalization. The difference in Vn values of V2/3 and V3/4 was noted in this work. The V2/3 values for all the samples (0.146), diploid rice (0.12), autotetraploid rice (0.135), PMA (0.119), MA (0.104), SCP (0.077) and BCP (0.106) samples were lower than 0.15 (Fig 5), which suggested that the top two reference genes are appropriate for the precise normalization of these development stages.

#### NormFinder analysis

The stability values of 14 candidate reference genes were calculated using NormFinder software, and lower values indicate higher stability of candidate reference genes and suitable for qRT-PCR analysis (Table 3). The most stable reference genes were *Cytochrome b5*, *CPI*, *GAPDH* and *PFP* in different comparison groups according to the NormFinder analysis. *Cytochrome b5* was the most stable gene in all total samples group, diploid, autotetraploid, and MA samples group, while *UBQ5* and *OsActin1* were the lowest stable reference genes in all the samples (Table 3). In different pollen development stages, *CPI* was the most stable reference gene in PMA samples (Table 3). *GAPDH* and *PFP* were found to be the most stable during PMA and BCP stages, respectively, and exhibited stability in other groups (Table 3). The stability classification of candidate reference genes produced by NormFinder program was different from that of GeNorm program for most of the samples.

**Table 3:** Stability analysis of candidate reference genes assayed by NormFinder software.

Rank	Total	Diploid	Autotetraploid	PMA	MA	SCP	BCP
1	*Cytochrome b5* (0.50)	*Cytochrome b5* (0.52)	*Cytochrome b5* (0.29)	*CPI* (0.39)	*Cytochrome b5* (0.64)	*GAPDH* (0.41)	*PFP* (0.13)
2	*CPI* (0.93)	*GAPDH* (0.79)	*CPI* (0.77)	*CPuORF7* (0.56)	*OsATG8a* (0.67)	*CPI* (0.42)	*OsATG8a* (0.16)
3	*SRP* (0.95)	*SRP* (0.79)	*OsATG8a* (0.77)	*SRP* (0.71)	*CPuORF7* (0.71)	*OsATG8a* (0.49)	*Cytochrome b5* (0.26)
4	*CPuORF7* (0.97)	*CPuORF7* (0.94)	*PFP* (0.83)	*Cytochrome b5* (0.73)	*CPI* (0.76)	*RIC1* (0.50)	*SRP* (0.47)
5	*GAPDH* (0.97)	*CPI* (0.97)	*CPuORF7* (0.93)	*PFP* (0.76)	*FLO16* (0.78)	*EF1a* (0.50)	*FLO16* (0.56)
6	*OsATG8a* (0.99)	*PFP* (1.05)	*GAPDH* (1.02)	*FLO16* (1.08)	*SRP* (0.79)	*Cytochrome b5* (0.51)	*RIC1* (0.66)
7	*PFP* (1.01)	*FLO16* (1.06)	*SRP* (1.10)	*GAPDH* (1.14)	*GAPDH* (0.91)	*PFP* (0.52)	*EF1a* (0.66)
8	*FLO16* (1.10)	*OsATG8a* (1.07)	*FLO16* (1.17)	*RIC1* (1.36)	*RIC1* (0.99)	*FLO16* (0.67)	*CPuORF7* (0.69)
9	*RIC1* (1.30)	*RIC1* (1.35)	*RIC1* (1.22)	*EF1a* (1.36)	*PFP* (1.05)	*SRP* (0.84)	*CPI* (0.70)
10	*EF1a* (1.31)	*EF1a* (1.38)	*EF1a* (1.22)	*OsATG8a* (1.48)	*EF1a* (1.10)	*OsActin1* (0.90)	*GAPDH* (0.86)
11	*OsAOC* (2.00)	*β-TUB* (1.68)	*OsAOC* (1.35)	*UBQ5* (1.62)	*OsAOC* (1.32)	*CPuORF7* (0.98)	*OsActin1* (1.05)
12	*β-TUB* (2.05)	*UBQ5* (2.50)	*OsActin1* (1.95)	*β-TUB* (1.81)	*β-TUB* (1.56)	*UBQ5* (1.17)	*UBQ5* (1.17)
13	*OsActin1* (2.37)	*OsAOC* (2.51)	*β-TUB* (2.01)	*OsActin1* (2.59)	*OsActin1* (2.25)	*β-TUB* (1.38)	*OsAOC* (1.48)
14	*UBQ5* (2.40)	*OsActin1* (2.72)	*UBQ5* (2.23)	*OsAOC* (3.32)	*UBQ5* (2.46)	*OsAOC* (1.45)	*β-TUB* (3.15)

Note: Total indicate the all samples of diploid and autotetraploid rice. Diploid indicate the all samples of diploid rice; Auto indicate the all samples of autotetraploid rice; PMA indicate the samples of pre-meiosis stage in diploid and autotetraploid rice; MA indicate the samples of meiosis stage in diploid and autotetraploid rice; SCP indicate the samples of single microspore stage in diploid and autotetraploid rice; BCP indicate the samples of bicellular pollen stage in diploid and autotetraploid rice.

#### BestKeeper analysis

The expression levels of 14 candidate reference genes were also assessed based on the Ct values using BestKeeper program (Table 4). Data with a SD < 1 exhibited acceptable variation, with lower SD and CV representing higher stability. Here, *Cytochrome b5* was the most stable gene for expression normalization in all the samples, group, and autotetraploid rice samples. *β-TUB* was the most stable in the diploid, MA, and SCP samples, and *PFP* was the most stable in PMA and BCP samples.

**Table 4:** Stability analysis of candidate reference genes assayed by BestKeeper software.

Rank	Total	Diploid	Autotetraploid	PMA	MA	SCP	BCP
1 CV±*SD*	*Cytochrome b5* 0.99±0.43	*β-TUB* 1.12±0.38	*Cytochrome b5* 0.64±0.23	*PFP* 0.46±0.18	*β-TUB* 1.08±0.36	*β-TUB* 0.89±0.28	*PFP* 0.33±0.13
2 CV±*SD*	*CPI* 1.08±0.46	*GAPDH* 1.23±0.57	*CPI* 0.92±0.40	*FLO16* 0.50±0.24	*OsATG8a* 1.10±0.45	OSACTIN1 0.91±0.65	*CPI* 0.42±0.20
3 CV±*SD*	*GAPDH* 1.14±0.53	*CPI* 1.29±0.56	*PFP* 0.98±0.37	*GAPDH* 0.64±0.31	*FLO16* 1.13±0.43	*OsAOC* 0.95±0.30	*OsATG8a* 0.44±0.19
4 CV±*SD*	*OsATG8a* 1.17±0.50	*FLO16* 1.32±0.52	*SRP* 1.01±0.35	*CPI* 0.65±0.29	*GAPDH* 1.15±0.53	*Cytochrome b5* 1.00±0.42	*Cytochrome b5* 0.45±0.19
5 CV±*SD*	*PFP* 1.24±0.47	*Cytochrome b5* 1.34±0.57	*OsATG8a* 1.03±0.44	*CPuORF7* 0.74±0.27	*Cytochrome b5* 1.15±0.49	*CPI* 1.02±0.43	*GAPDH* 0.46±0.23
6 CV±*SD*	*SRP* 1.28±0.46	*CPuORF7* 1.35±0.50	*GAPDH* 1.05±0.50	*Cytochrome b5* 0.84±0.38	*CPI* 1.18±0.50	*RIC1* 1.02±0.35	*FLO16* 0.47±0.20
7 CV±*SD*	*FLO16* 1.34±0.53	*OsATG8a* 1.37±0.58	*RIC1* 1.11±0.37	*OsATG8a* 0.93±0.41	*CPuORF7* 1.26±0.45	*EF1a* 1.02±0.38	*SRP* 0.49±0.18
8 CV±*SD*	*CPuORF7* 1.39±0.51	*PFP* 1.50±0.56	*CPuORF7* 1.11±0.40	*SRP* 0.98±0.36	*OsActin1* 1.26±1.00	*GAPDH* 1.03±0.46	*CPuORF7* 0.55±0.21
9 CV±*SD*	*RIC1* 1.41±0.47	*SRP* 1.55±0.56	*EF1a* 1.11±0.37	*RIC1* 1.34±0.46	*PFP* 1.35±0.50	*SRP* 1.11±0.40	*RIC1* 0.61±0.20
10 CV±*SD*	*EF1a* 1.45±0.48	*RIC1* 1.72±0.57	*FLO16* 1.33±0.52	*EF1a* 1.37±0.56	*SRP* 1.38±0.48	*PFP* 1.13±0.40	*EF1a* 0.62±0.25
11 CV±*SD*	*β-TUB* 1.54±0.50	*EF1a* 1.79±0.60	*OsAOC* 1.40±0.43	*β-TUB* 1.74±0.60	*OsAOC* 1.41±0.43	*OsATG8a* 1.18±0.50	*UBQ5* 1.07±3.22
12 CV±*SD*	*OsAOC* 1.82±0.57	*UBQ5* 1.89±0.64	*β-TUB* 1.56±0.50	*UBQ5* 1.82±0.62	*RIC1* 1.55±0.50	*FLO16* 1.38±0.51	*OsActin1* 1.10±0.67
13 CV±*SD*	*OsActin1* 1.86±0.13	*OsActin1* 2.04±1.54	*OsActin1* 1.59±1.11	*OsActin1* 2.36±1.76	*EF1a* 1.67±0.54	*UBQ5* 1.42±0.45	*OsAOC* 1.21±0.37
14 CV±*SD*	*UBQ5* 2.09±0.69	*OsAOC* 2.29±0.72	*UBQ5* 1.84±0.57	*OsAOC* 2.86±0.95	*UBQ5* 1.94±0.67	*CPuORF7* 1.51±0.54	*β-TUB* 2.57±0.81

Note: Total indicate the all samples of diploid and autotetraploid rice. Diploid indicate the all samples of diploid rice; Auto indicate the all samples of autotetraploid rice; PMA indicate the samples of pre-meiosis stage in diploid and autotetraploid rice; MA indicate the samples of meiosis stage in diploid and autotetraploid rice; SCP indicate the samples of single microspore stage in diploid and autotetraploid rice; BCP indicate the samples of bicellular pollen stage in diploid and autotetraploid rice.

Consistent with the ΔCt method and NormFinder analysis, *Cytochrome b5* was the most stable gene in all the samples, especially in autotetraploid rice samples. However, the stability classification of the candidate reference genes produced with PMA, MA, SCP and BCP was different from that of ΔCt method and Normfinder analysis in most of the samples (Table 4). For example, *PFP* and *β-TUB* were identified as the most stable reference genes in the PMA, MA and SCP stage in the BestKeeper analysis, while their stability rankings were fourth and third in PMA and MA stage with the ΔCt method analysis, respectively.

#### RefFinder analysis

The comprehensive rankings of the candidate reference genes were determined with the RefFinder program, which integrates different analysis programs, including BestKeeper, NormFinder, GeNorm and ΔCt method analysis (Table 5). *PFP*, *Cytochrome b5* and *CPI* were found to be the most stable reference genes in all the samples (Table 5). *Cytochrome b5* were suggested to be the most suitable reference gene for all differential groups, such as total number of samples, diploid, autotetraploid, and MA samples (Table 5). The most suitable combination for the samples was *Cytochrome b5* together with *CPI*. *UBQ5* or *OsActin1* was the least stable reference gene under the majority of different pollen development stages.

**Table 5:** Stability analysis of candidate reference genes assayed by RefFinder software.

Rank	Total	Diploid	Autotetraploid	PMA	MA	SCP	BCP
1	*Cytochrome b5* (1.41)	*Cytochrome b5* (1.48)	*Cytochrome b5* (1.41)	*PFP* (2.11)	*Cytochrome b5* (1.41)	*CPI* (1.78)	*PFP* (1.78)
2	*CPI* (1.68)	*SRP* (1.49)	*CPI* (2.91)	*CPuORF7* (2.51)	*OsATG8a* (2.38)	*Cytochrome b5* (2.91)	*Cytochrome b5* (2.63)
3	*GAPDH* (2.94)	*GAPDH* (1.51)	*OsATG8a* (3.50)	*CPI* (2.63)	*FLO16* (2.94)	*GAPDH* (2.99)	*OsATG8a* (3.46)
4	*SRP* (4.56)	*CPuORF7* (1.53)	*PFP* (4.43)	*FLO16* (3.03)	*CPI* (4.68)	*OsATG8a* (5.07)	*SRP* (3.98)
5	*OsATG8a* (4.68)	*CPI* (1.54)	*RIC1* (4.90)	*Cytochrome b5* (4.36)	*SRP* (5.96)	*PFP* (5.38)	*RIC1* (4.05)
6	*PFP* (5.01)	*FLO16* (1.57)	*SRP* (4.92)	*GAPDH* (4.41)	*CPuORF7* (5.96)	*RIC1* (5.63)	*EF1a* (4.53)
7	*CPuORF7* (6.29)	*OsATG8a* (1.60)	*EF1a* (5.33)	*SRP* (5.38)	*β-TUB* (6.45)	*EF1a* (6.19)	*CPI* (6.18)
8	*FLO16* (7.20)	*PFP* (1.61)	*CPuORF7* (5.92)	*RIC1* (8.49)	*GAPDH* (6.59)	*β-TUB* (6.85)	*FLO16* (6.19)
9	*RIC1* (9.00)	*RIC1* (1.82)	*GAPDH* (6.64)	*EF1a* (8.97)	*PFP* (6.64)	*OsActin1* (6.85)	*CPuORF7* (8.00)
10	*EF1a* (10.00)	*EF1a* (1.84)	*FLO16* (9.46)	*OsATG8a* (9.15)	*RIC1* (8.56)	*SRP* (8.74)	*GAPDH* (8.41)
11	*OsAOC* (11.24)	*β-TUB* (2.25)	*OsAOC* (11.00)	*UBQ5* (11.24)	*EF1a* (10.40)	*FLO16* (9.12)	*OsActin1* (11.24)
12	*β-TUB* (11.74)	*UBQ5* (2.80)	*OsActin1* (12.24)	*β-TUB* (11.74)	*OsAOC* (11.00)	*OsAOC* (9.53)	*UBQ5* (11.74)
13	*OsActin1* (13.00)	*OsAOC* (2.86)	*β-TUB* (12.74)	*OsActin1* (13.00)	*OsActin1* (11.51)	*CPuORF7* (11.41)	*OsAOC* (13.00)
14	*UBQ5* (14.00)	*OsActin1* (2.98)	*UBQ5* (14.00)	*OsAOC* (14.00)	*UBQ5* (14.00)	*UBQ5* (12.24)	*β-TUB* (14.00)

Note: Total indicate the all samples of diploid and autotetraploid rice. Diploid indicate the all samples of diploid rice; Auto indicate the all samples of autotetraploid rice; PMA indicate the samples of Pre-meiosis stage in diploid and autotetraploid rice; MA indicate the samples of meiosis stage in diploid and autotetraploid rice; SCP indicate the samples of single microspore stage in diploid and autotetraploid rice; BCP indicate the samples of bicellular pollen stage in diploid and autotetraploid rice.

### Verification of selected reference genes in autotetraploid rice

**Fig 6:**
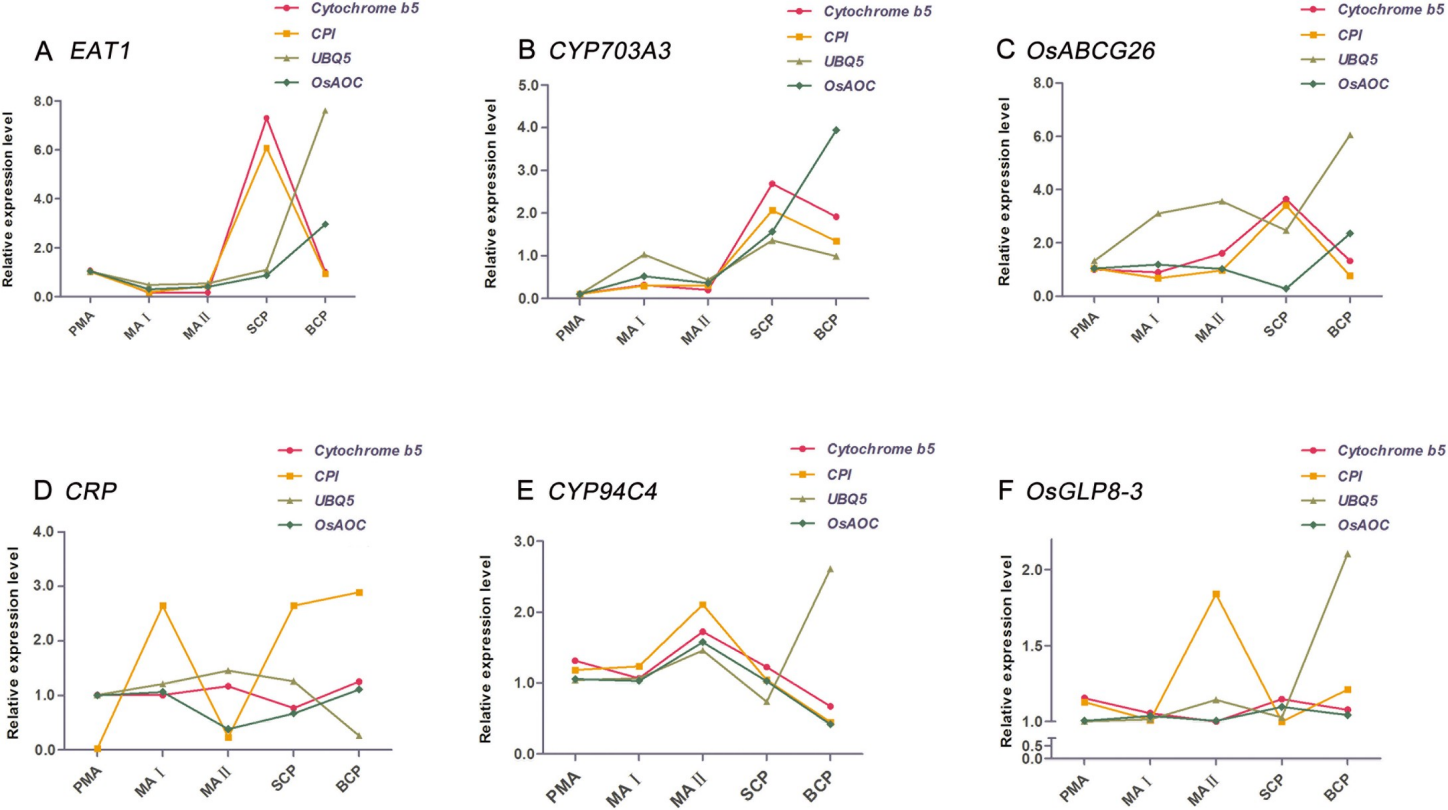
Relative expression of pollen development related and stage specific genes using the selected reference genes for normalization. (A) The expression of *EAT1* in different pollen development stages was normalized using the most stable genes and least stable reference genes. (B) The expression of *CYP703A3* in different pollen development stages was normalized using the two most stable genes and least stable reference genes. (C) The expression of *OsABCG26* in different pollen development stages was normalized using the most stable genes and least stable reference genes. (D) The expression of *CRP* in different pollen development stages was normalized using the most stable genes and least stable reference genes. (E) The expression of *CYP94C4* in different pollen development stages was normalized using the most stable genes and least stable reference genes. (F) The expression of *OsGLP8-3* in different pollen development stages was normalized using the most stable genes and least stable reference genes (*UBQ5* and *OsActin1*). PMA indicate the samples of pre-meiosis stage in diploid and autotetraploid rice; MAI indicate the samples of meiosis stage I in diploid and autotetraploid rice; MAII indicate the samples of meiosis stage II in diploid and autotetraploid rice; SCP indicate the samples of single microspore stage in diploid and autotetraploid rice; BCP indicate the samples of bicellular pollen stage in diploid and autotetraploid rice.

To find most or least stable gene as described above, we selected two most stable references genes (*Cytochrome b5* and *CPI*) and the two least stable reference genes (*UBQ5* and *OsActin1*) to verify the expression levels of six target genes in Taichung65-4x (Fig 6, S4 and S5 Figs in S1 File). Among these genes, *EAT1*, *CYP703A3* and *OsABCG26* are pollen development related genes. *EAT1* is a helix-loop-helix DNA-binding domain containing protein and regulate the tapetal programmed cell death during male reproduction. *OsABCG26* is an ATP binding cassette G transporter, and play the important role in male reproduction process. *CYP703A3* is a cytochrome P450 and play vital role in pollen exine and anther cuticle development. We conducted the qRT-PCR analysis to measure transcript levels of three pollen development related gene (*EAT1*, *CYP703A3* and *OsABCG26*) using the two stable (*Cytochrome b5* and *CPI*) and unstable reference genes (*UBQ5* and *OsActin1*) (Fig 6A–6C). Using the *Cytochrome b5* or *CPI* alone or *Cytochrome b5* combined with *CPI*, we found that transcript levels of pollen development related genes detected were consistent with the transcriptome data and much more different from the transcript levels of unstable reference genes (*UBQ5* and *OsActin1*).

In addition, we also selected three pollen development stage specific genes *CRP*, *CYP94C4*, and *OsGLP8-3* and conducted the qRT-PCR analysis in autotetraploid rice (Fig 6D–6F). Using the *Cytochrome b5* or *CPI* alone or *Cytochrome b5* combined with *CPI* as the reference gene, we found that transcript levels of pollen development stage specific genes were consistent with the transcriptome data and much more different from the transcript levels of *UBQ5* and *OsActin1* (Fig 6D–6F). All of these results indicated that reference genes selected from transcriptome data by five different algorithms are reliable.

## Discussion

### Significant phenotypic variations detected in autotetraploid rice compared to diploid rice

Autotetraploid rice is a newly developed polyploid material through chromosome doubling by colchicine treatment. In contrast with diploid progenitors, autotetraploid rice have greater genetic variation in agronomic traits, such as varying seed set along with longer grains and awns [3, 30, 31]. Moreover, autotetraploid rice showed great potential to increase the yield of rice, and high nutrition and resistant to insect pests and diseases [3, 30]. Low pollen fertility is the primary hindrance in the utilization of autotetraploid rice [4–6]. Therefore, it is critical to reveal the reasons for low pollen fertility in autotetraploid rice.

Autotetraploid rice with doubling of chromosomes displayed genetic variations at morphological, cytological and molecular level [1, 3, 32]. In the present work, we performed the phenotypic analysis for a better understanding of phenotypic variations between Taichung65-4x and its corresponding diploid rice. Our results indicated that autotetraploid rice Taichung65-4x had higher grain width, and grain length, but lower seed set, and number of effective panicles per plant. These results in agreement with our previous studies, which showed significant variations in agronomic traits in tetraploid rice compared to diploid counter parts [1, 7, 30]. Cytological observations were further conducted to observe the pollen development process between Taichung65-4x and its corresponding diploid rice. Here, low pollen fertility and different types of abnormalities were detected in Taichung65-4x compared with Taichung65-2x. Cell shrinkage, cell degeneration, callose without disassembly and irregular-shaped cells were frequently detected in Taichung65-4x compared with diploid rice. Additionally, anther structure and abnormal PMCs were also observed in Taichung65-4x. All of these results indicated remarkable phenotypic variations in autotetraploid rice compared to diploid rice.

### Selection of candidate reference genes is indispensable to analyze the gene expression patterns in autotetraploid rice

Quantitative reverse transcription PCR (qRT-PCR) is a commonly used method and has become the primary quantitative technique for precise expression profiling of target genes. Several factors affect the accuracy of qRT-PCR, including reverse transcription and PCR amplification efficiency, RNA integrity and sample quality, cDNA concentration, and technical related factors [10, 33, 34]. To achieve the reliable gene expression results, it is essential to identify the stable reference genes for the gene expression analysis. A large number of studies, which intended to detect javascript:;reference genes as internal controls for qRT-PCR analysis, have been conducted in rice [14, 22, 35]. *Eif-4a* is a stable reference gene for rice endosperm development, but its expression is unstable under high temperature [36]. *EF1a* and *UBQ5* displayed the most stable expression levels in 25 rice samples that were isolated from various tissues at different development stages [14, 15]. However, glycine-rich RNA-binding protein gene (GBP) was also reported as the most stable gene for the whole growth period in rice [37]. Till now, no universal reference gene has been reported that could be used for all rice tissues and/or experimental conditions [38–40].

The reliability of reference genes depends on stability and estimating the expression levels of a target gene. The selection of reliable reference gene is vital for qRT-PCR analysis, but no single reference gene is appropriate for all plant species, development stage, tissues or experimental conditions. Hence, it is important to assess the stability of candidate reference gene under different experimental conditions [22, 41]. In the previous studies, candidate reference genes were mainly selected from conventionally used housekeeping genes or their homologs [14, 42]. Recently, systemic transcriptome analyses, including RNA-sequencing and DNA microarray were also extensively used in the study of the rice development process. The publicly available transcriptome data can be used to select proper reference genes for different development stages [22, 37]. Transcriptome analysis was found to be an effective tool to investigate the pollen development process in autotetraploid rice [7, 43–45], which have provided the possibility to select the new candidate genes for autotetraploid rice or polyploidy effect by the analysis of gene expression patterns across various experimental conditions.

From this study, 14 candidate reference genes were selected to evaluate their stability in pollen development between autotetraploid and diploid rice. Of these genes, seven were commonly used housekeeping genes and other seven genes were selected from the transcriptome data of diploid and autotetraploid rice as candidate reference genes. The commonly employed reference genes such as, *GAPDH*, *OsActin1*, *UBQ5* and *18s rRNA* were frequently used and their expression levels varied considerably in different tissues [14]. The expression levels of these genes (with more uniform expression patterns) can be used for normalization of real-time PCR results for gene expression studies in a wide range of rice samples. *UBQ5* and *eEF-1a* were the most stable reference genes selected from 10 commonly used genes in a diverse set of 25 rice samples [14]. In the present work, we also used *UBQ5* and *eEF-1a* as candidate reference genes and found that expression patterns of common housekeeping reference genes were varied in pollen development stages and different polyploidy level. For example, *UBQ5* was the most stable reference gene in diploid rice pollen development stage in a previous study [14, 15]. However, *UBQ5* was the least stable reference gene during different pollen development stages in diploid and autotetraploid rice. Here, *Cytochrome b5* was found to be the most stable reference gene in most of comparison groups compared with the commonly used candidate reference genes. Our results indicated that candidate reference genes selected from transcriptome data were more stable than the commonly used reference genes.

In the present work, we also found that *UBQ5* and *OsActin1* displayed very high expression levels (i.e. very low Ct values) but was classified as one of the most unstable candidate genes by the five algorithms accessed by RefFinder. The reason for this phenomenon is that the criterion for selection of a reference gene involves the gene expression patterns at a moderate level [23]. In addition, the protein coded by *UBQ5* and *OsActin1* takes part in alternative metabolic roles, therefore high variability was detected in expression levels. Instability in the expression patters of *UBQ5* and *OsActin1* have been found in rice, although genes showed stable expression levels in other organs/tissues of rice [15, 36]. The above results revealed that candidate reference genes based on transcriptome are more stable than the common reference genes.

### Selection of the stable candidate reference genes using different methods in autotetraploid rice

To ensure the stability of reference genes in autotetraploid rice and diploid rice during pollen development, we analyzed the normalization of gene expression data using different statistical methods. Here, we have used five statistical programs, namely NormFinder [25], delta Ct (ΔCt) method [11], BestKeeper [12], geNorm [11] and RefFinder, which have been frequently used by researchers [22, 37, 41]. A total of 14 candidate reference genes were tested for the detection of suitable reference genes for autotetraploid rice during different pollen development stages. The results indicated that order of the best or worst candidate reference genes varied slightly among different stages. For example, RefFinder exhibited *Cytochrome b5*, *PFP*, and *CPI* as the most stable references and *UBQ5*, *OsAOC* and *β-TUB* as the three least stable genes among the 14 candidate reference genes. Although candidate reference genes, such as *UBQ5*, *OsAOC* and *β-TUB* were applied frequently as reference genes for normalization in the gene expression analysis of different rice development stages. These results showed that candidate reference genes should be assessed prudently by combining two algorithm or more than two validation methods, which can yield variable results.

To validate the stability of candidate reference genes, *Cytochrome b5*, *CPI*, *UBQ5* and *OsActin1*, were selected for normalization of raw qRT-PCR data of six pollen development related genes as target genes in different pollen development stages. From this study, similar expression patterns were detected, when two stable reference genes (*Cytochrome b5* and *CPI*) were tested for normalization. In contrast, when the least stable genes (*UBQ5* and *OsActin1*) were tested for normalization, the different results were obtained. The expression patterns of six pollen development related genes indicated an exponential difference when the qRT-PCR data was normalized with the least and most stable reference genes. Therefore, the selection of reference genes can directly affect the expression levels of target genes.

In summary, we conducted and established the effective method to select stable reference genes for autotetraploid rice, especially to evaluate the polyploidy effect. The reference genes detected by using transcriptome analysis, *Cytochrome b5* and *CPI*, displayed less spatio-temporal specificity and are appropriate for different pollen development stages than commonly housekeeping genes, which could be used as ideal reference genes for pollen development in polyploid plants.

## Supporting information

S1 File Additional figures and tables about pollen development, morphological traits and gene expression in autotetraploid and diploid rice. (PDF)

S1 Raw images (PDF)

## List of abbreviations

qRT-PCR: Quantitative reverse transcription PCR
TIGR: Rice genome annotation project database
GEO: Gene expression omnibus database
Rice XPro: Rice expression profile database
WE-CLSM: Whole-mount eosin B-staining confocal laser scanning microscopy
Ct: cycle threshold
CV: coefficients of variation
SD: standard deviation
PMA: pre-meiosis stage
MA: meiosis stage
SCP: single microspore stage
BCP: bicellular pollen stage
