# Supplementary material for: Identification of stable pollen development related reference genes for accurate qRT-PCR analysis and morphological variations in autotetraploid and diploid rice
Source: PLoS One. 2021 Jun 29;16(6):e0253244. doi: 10.1371/journal.pone.0253244 (PMC8241056; doi:10.1371/journal.pone.0253244)
Supplement: S1 File — (PDF) [file pone.0253244.s001.pdf]

Supporting Information

S1 File. Additional figures and tables about pollen development, morphological traits and gene expression in autotetraploid and diploid rice

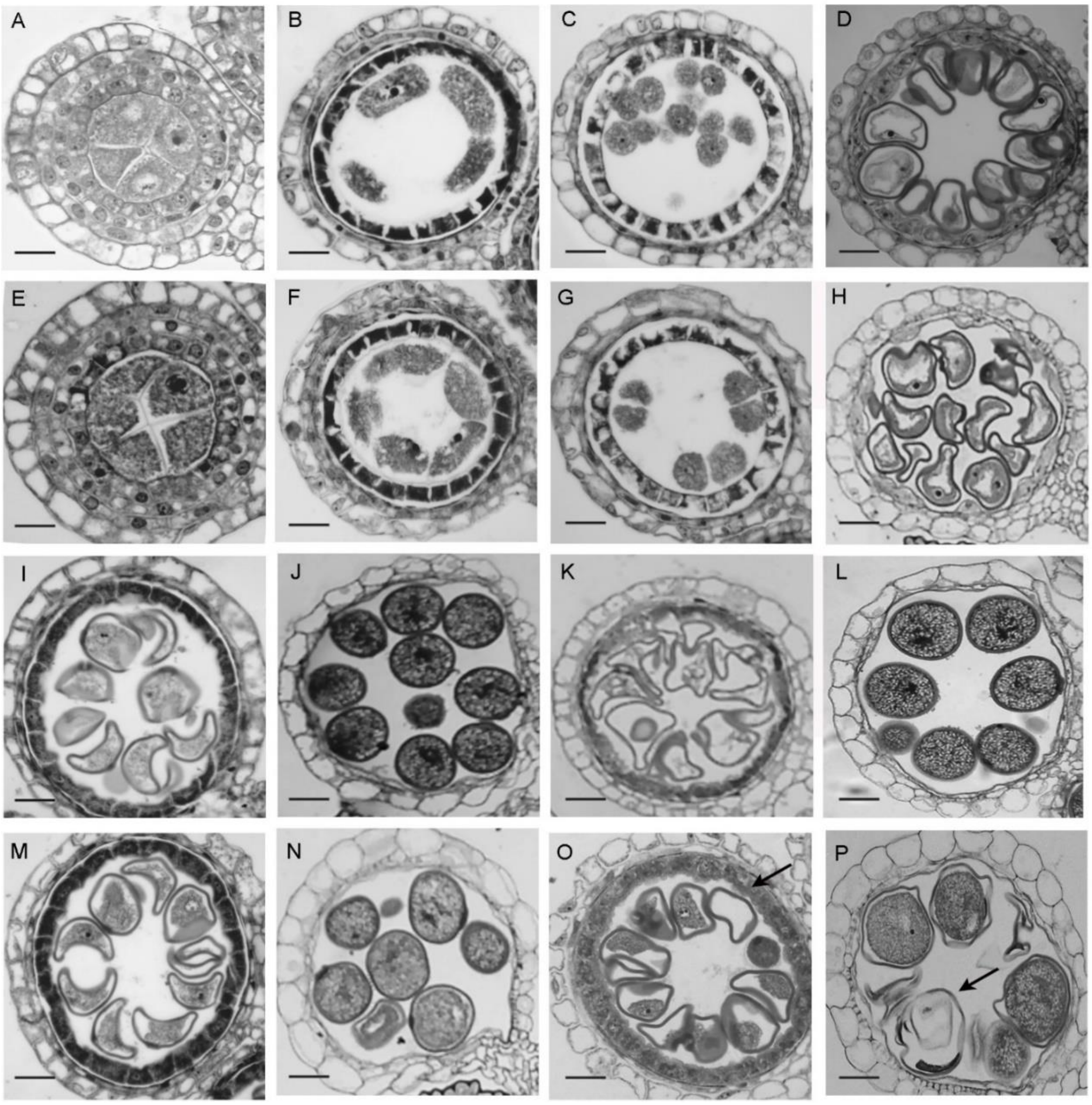

S1 Fig. Semi-thin sections of Taichung65-4x and its corresponding diploid rice.

Note：(A-D), (I-L) Semi-thin sections of Taichung65-2x anthers. (A) pre-meiosis stage; (B) meiosis stage I; (C) meiosis stage II; (D) single microspore stage; (I) middle bicellular stage; (J) mature pollen stage; (K) single microspore stage; (L) mature pollen stage. (E-H), (M-P) Semi-thin sections of Taichung65-4x anthers. (E) pre-meiosis stage; (F) meiosis stage I; (G) meiosis stage II; (H) single microspore stage; (M) middle bicellular stage; (N) mature pollen stage; (O) single microspore stage, abnormal (arrow); (P) mature pollen stage, typical abortion (arrow). Bars = 50 μm.

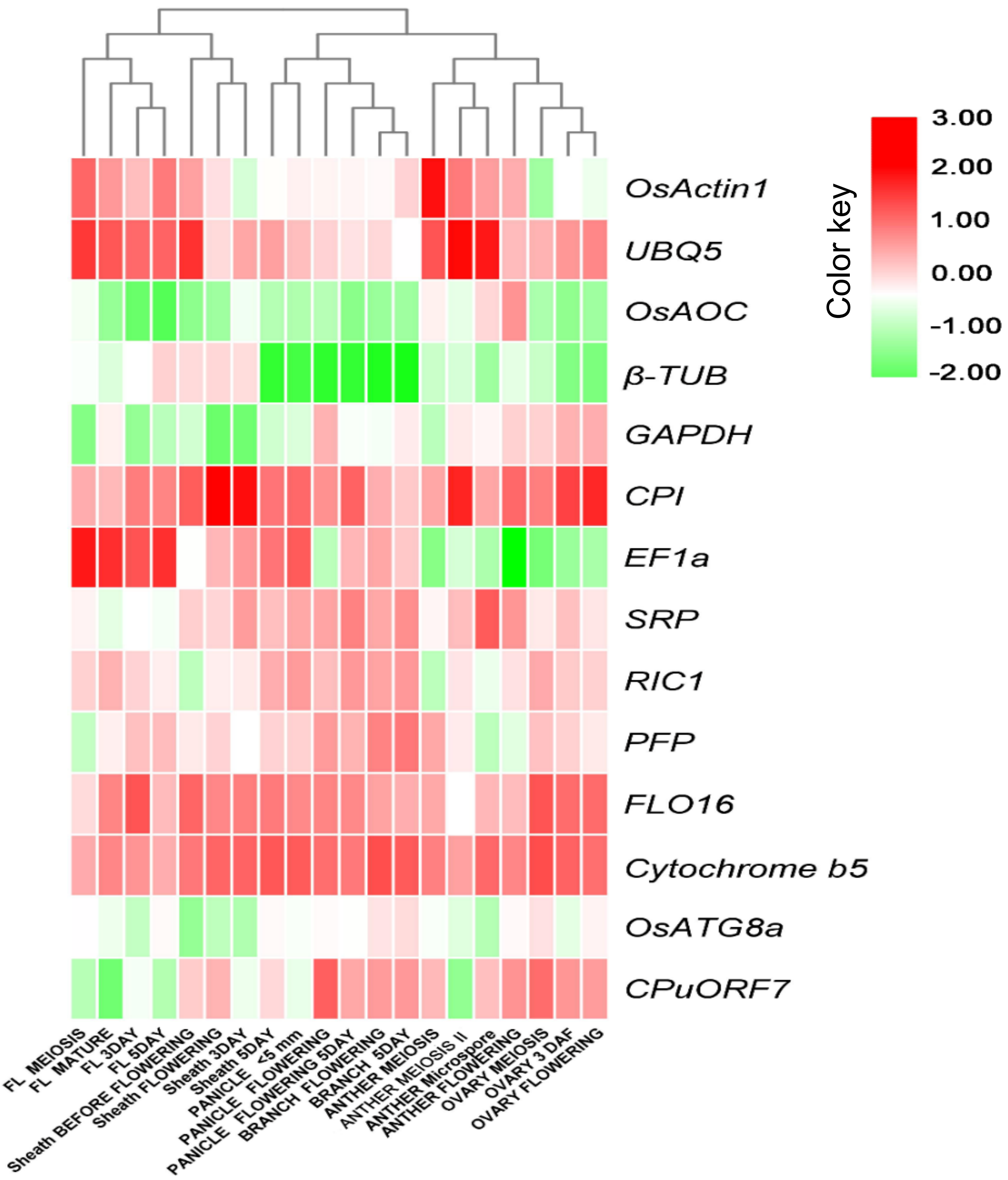

S2 Fig. Heat map of expression of 14 candidate reference genes using transcriptome data from various pollen development stage in autotetraploid rice.

Note: Green indicates low transcript levels and red indicates high transcript levels.

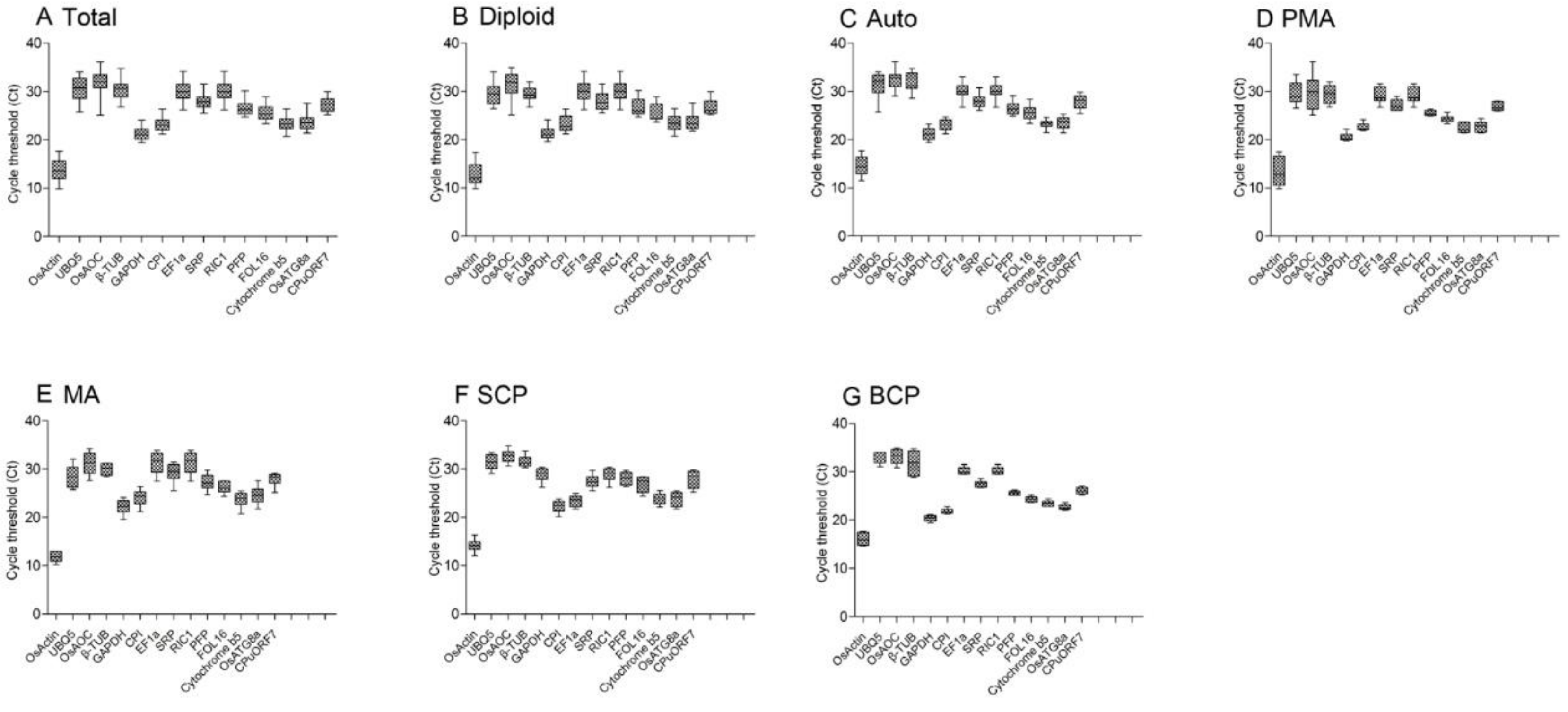

S3 Fig. Expression levels of 14 candidate reference genes in different comparison groups.

The variability of the Ct values of each reference gene is shown in scatter graphs. (A) Total samples of autotetraploid rice and its corresponding diploid rice. (B) All samples of diploid rice. (C) All samples of autotetraploid rice. (D) Samples of pre-meiosis stage in autotetraploid and diploid rice. (E) Samples of meiosis stage in autotetraploid and diploid rice. (F) Samples of single microspore stage in autotetraploid and diploid rice. (G) Samples of bicellular pollen stage in autotetraploid and diploid rice. Total indicate the all samples of diploid and autotetraploid rice. Diploid indicate the all samples of diploid rice; Auto indicate the all samples of autotetraploid rice; PMA indicate the samples of pre-meiosis stage in diploid and autotetraploid rice; MA indicate the samples of meiosis stage in diploid and autotetraploid rice; SCP indicate the samples of single microspore stage in diploid and autotetraploid rice; BCP indicate the samples of bicellular pollen stage in diploid and autotetraploid rice.

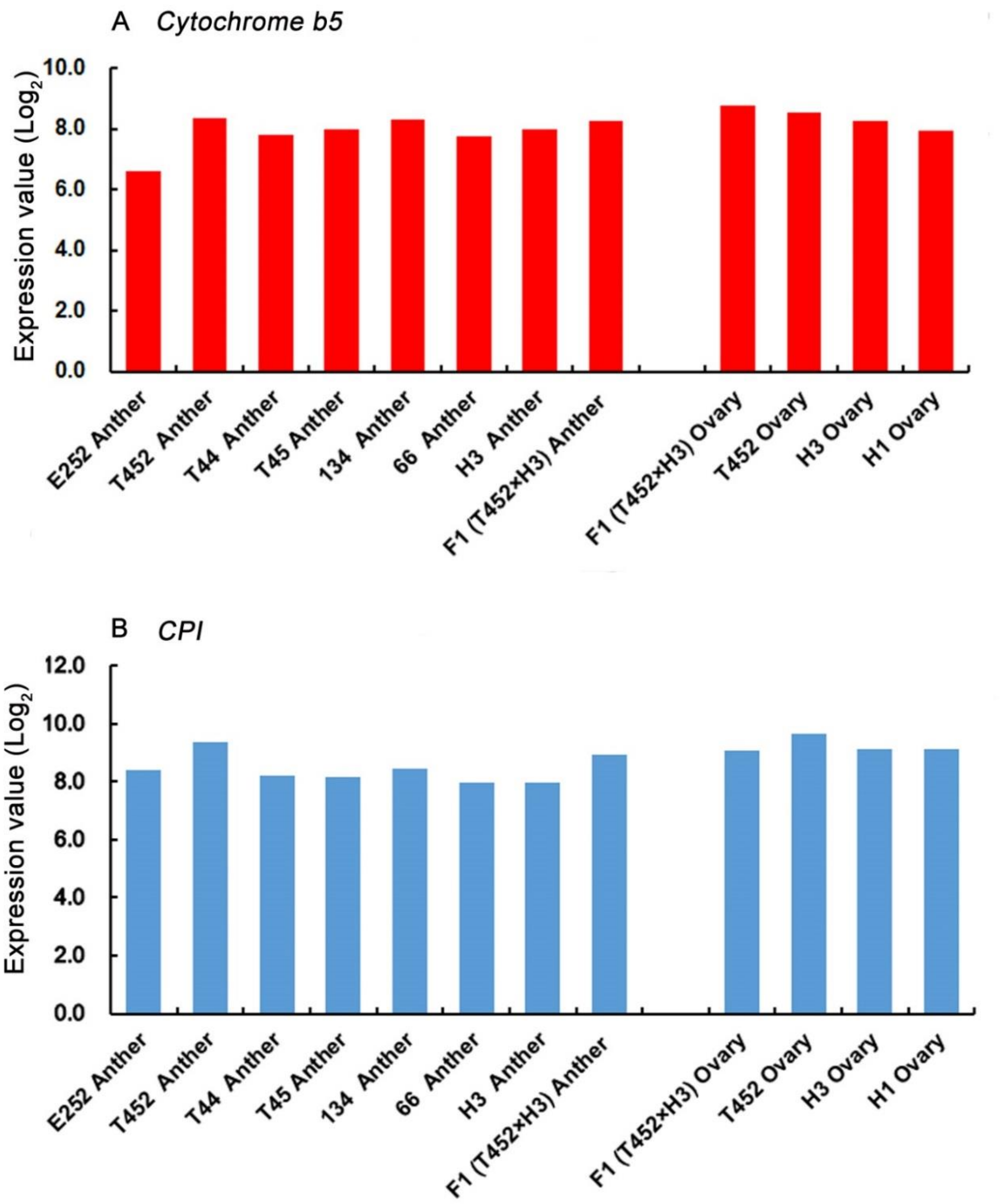

S4 Fig. Expression values of two candidate reference genes using transcriptome data from various pollen development stage in different types of rice.

Note: E252 indicated the diploid rice; T452, T44, T45 indicated the autotetraploid rice; 134,66, H3, H1 indicated the neo-tetraploid rice; F_1 (T452×H3) indicated the hybrid of tetraploid rice

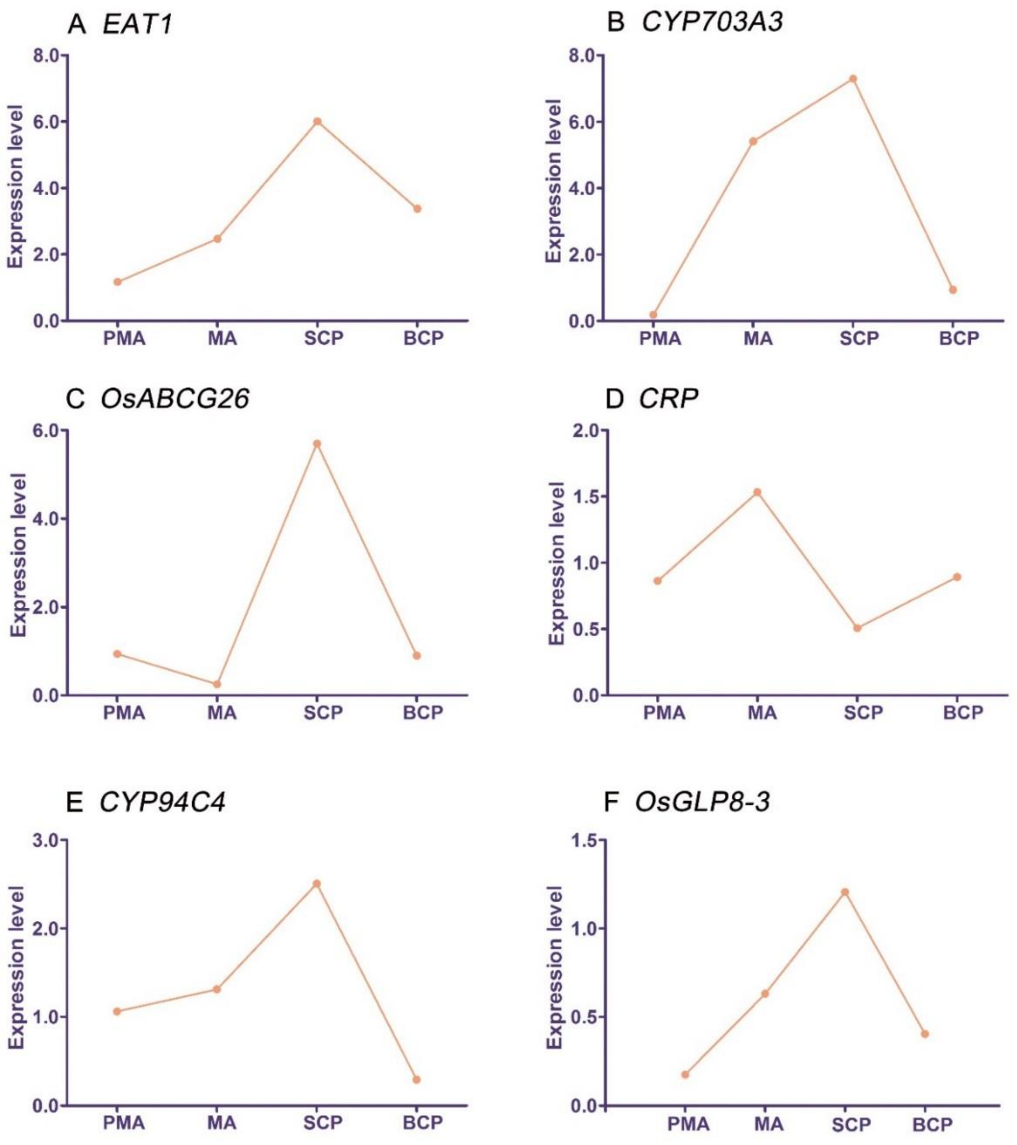

S5 Fig. The transcript levels of selected genes in autotetraploid rice during different pollen development stages.

Note: (A) The transcript levels of *EAT1* in different pollen development stages; (B) The transcript levels of *CYP703A3* in different pollen development stages; (C) The transcript levels of *OsABCG26* in different pollen development stages; (D) The transcript levels of *CRP* in different pollen development stages; (E) The transcript levels of *CYP94C4* in different pollen development stages; (F) The transcript levels of *OsGLP8-3* in different pollen development stages.

S1 Table. Floret length in Taichung65-4x and its corresponding diploid rice during pollen development stages.

Floret length	Taichung65-2x	Taichung65-4x
PMA	2.5-3.0 mm	3.5-4.0 mm
MA	3.5-4.5 mm	4.5-5.5 mm
SCP	5.5-6.0 mm	6.5-7.0 mm
BCP	6.5-7.0 mm	7.0-7.5 mm

Note: PMA indicate the pre-meiosis stage in diploid and autotetraploid rice; MA indicate the meiosis stage in diploid and autotetraploid rice; SCP indicate the single microspore stage in diploid and autotetraploid rice; BCP indicate the bicellular pollen stage in diploid and autotetraploid rice.

S2 Table. Comparison of morphological traits in Taichung65-4x and its corresponding diploid rice

**Traits**	**Taichung65-2x**	**Taichung65-4x**
Plant height (cm)	122.00±1.13	110.40±1.54**
Number of effective panicles	9.25±1.25	5.20±0.65**
Flag leaf length (cm)	39.30±1.50	34.10±1.17**
Flag leaf width (cm)	1.80±0.12	1.70±0.02
Panicle length（cm）	21.62±0.17	24.10±0.08
Seed set (%)	82.10±1.10	40.40±2.16**
Grain length (cm)	0.73±0.01	0.84±0.04
Grain width (cm)	0.33±0.12	0.37±0.01
Grain length to width ratio	2.21±0.02	2.27±0.03

**Significant difference at $p < 0.01$

S3 Table. Expression levels of 14 candidate reference genes

	OsActin1	***UBQ5***	***OsAOC***	***β-TUB***	***GAPDH***	***EF1a***	***CPI***	***SRP***	***RIC1***	***PFP***	***FLO16***	***Cytochrome b5***	***OsATG8a***	***CPuORF7***
Diploid	13.07	29.57	31.35	29.46	21.47	30.08	23.17	27.90	30.17	26.73	25.43	23.40	23.76	26.75
Auto	14.43	31.53	32.37	31.73	21.24	30.05	23.02	28.09	30.05	26.50	25.70	23.38	23.55	27.88
PMA	13.38	29.58	29.84	29.47	20.53	29.17	22.61	27.15	29.17	25.50	24.31	22.38	22.75	26.85
MA	12.60	29.25	31.80	30.05	21.77	31.06	23.81	28.91	31.16	26.93	26.12	23.61	24.50	27.87
SCP	14.13	31.46	32.73	31.56	22.31	28.88	23.41	27.47	28.88	28.07	26.96	23.87	23.85	27.96
BCP	16.04	33.20	33.14	31.84	20.41	30.16	21.82	27.53	30.16	25.65	24.31	23.47	22.64	26.05
AV	13.75	30.55	31.86	30.59	21.36	30.07	23.09	27.99	30.11	26.62	25.56	23.39	23.65	27.32
SD	2.22	2.48	2.40	1.96	1.35	1.86	1.35	1.58	1.83	1.55	1.58	1.28	1.48	1.53
CV (%)	16.11	8.12	7.53	6.41	6.31	6.17	5.86	5.65	6.07	5.81	6.18	5.48	6.25	5.58

Note: Diploid indicate the total samples of diploid rice; Auto indicate the total samples of autotetraploid rice; PMA indicate the Pre-meiosis stage in diploid and autotetraploid rice; MA indicate the meiosis stage in diploid and autotetraploid rice; SCP indicate the single microspore stage in diploid and autotetraploid rice; BCP indicate the bicellular pollen stage in diploid and autotetraploid rice.
