# Supplementary material for: Identification of stable pollen development related reference genes for accurate qRT-PCR analysis and morphological variations in autotetraploid and diploid rice
Source: PLoS One. 2021 Jun 29;16(6):e0253244. doi: 10.1371/journal.pone.0253244 (PMC8241056; doi:10.1371/journal.pone.0253244)
Supplement: S1 Raw images — (PDF) [file pone.0253244.s002.pdf]

S1 Raw Images

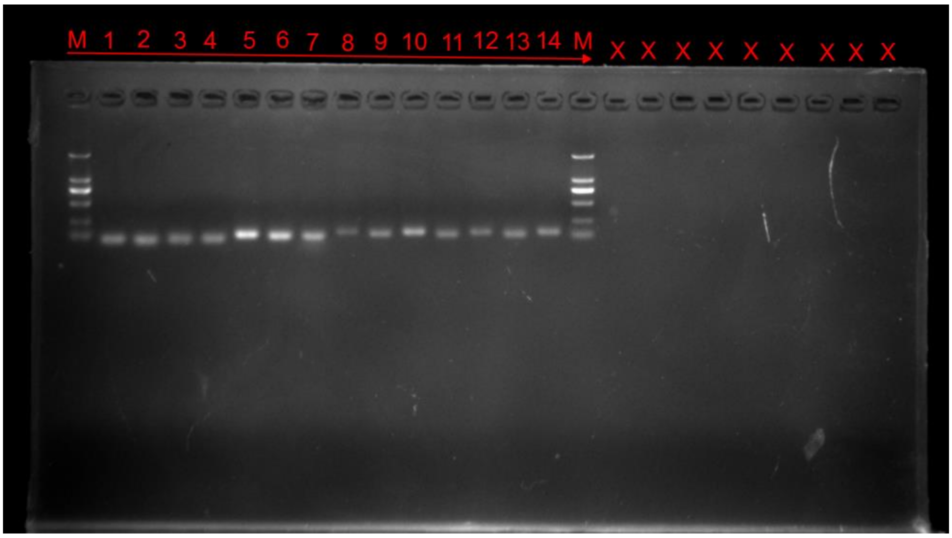

Following are three different original gel images of same figure

Fig 3. Specificity of primers and its amplification size. (A) Amplification of PCR products for 14 genes in agarose gel (1.5%) electrophoresis. (M: DNA Marker 2000bp; lanes 1-14: *OsActin1*, *UBQ5*, *OsAOC*, *β-TUB*, *GAPDH*, *CPI*, *EF-1a*, *SRP*, *RIC1*, *PFP*, *FLO16*, *Cytochrome b5*, *OsATG8a* and *CPuORF7*, respectively.

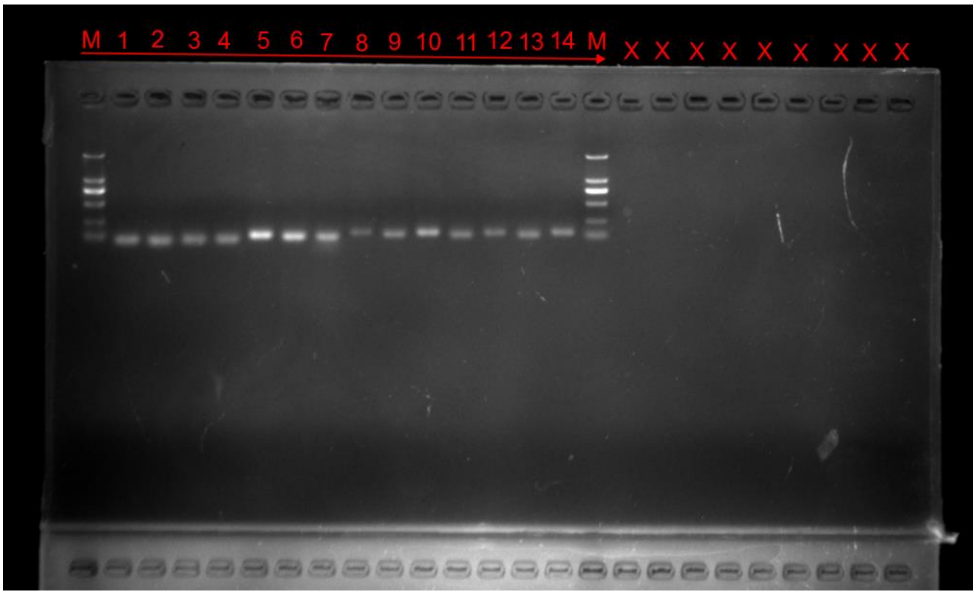

Same Figure, but another irrelevant gel can also be seen

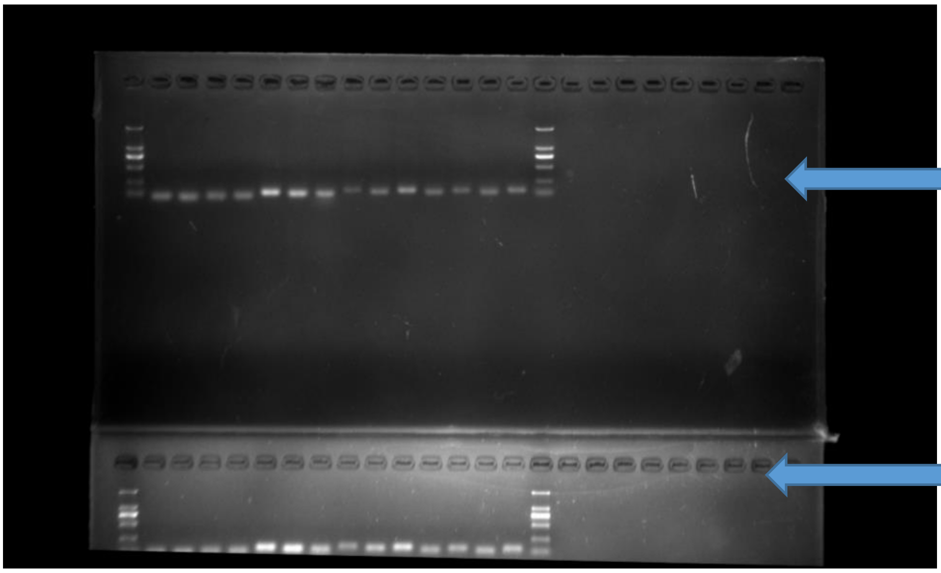

Only First Pic is used in this study.

This Gel is not relevant to this manuscript
